# Night Shifts and Occupational Fatigue Among Nurses Who Work 12-Hour-Shifts in Jeddah

**DOI:** 10.3390/healthcare14203371

**Published:** 2026-10-09

**Authors:** Bayan Alanmi, Ruba Alharazi, Hayfa Almutary

**Affiliations:** Department of Medical Surgical Nursing, Faculty of Nursing, King Abdulaziz University, Jeddah 21589, Saudi Arabia; balanmi0001@stu.kau.edu.sa (B.A.); aalalmetere2@kau.edu.sa (H.A.)

**Keywords:** night shift, nurses, occupational fatigue, 12 h shifts, intershift recovery, Saudi Arabia

## Abstract

**Highlights:**

**What are the main findings?**

- Night-shift work had substantial physiological, psychological, and social impacts among nurses working 12 h shifts.
- Greater night-shift impacts were associated with higher chronic and acute fatigue and poorer intershift recovery.

**What are the implications of the main findings?**

- Balanced night shift scheduling and adequate recovery periods may help reduce occupational fatigue among nurses.
- Healthcare organizations should strengthen workplace support and fatigue-management strategies for nurses working 12 h night shifts.

**Abstract:**

**Background/Objectives**: Night-shift work is essential for continuous nursing care but may disrupt circadian rhythms, impair well-being, and contribute to occupational fatigue. This study assessed the impact of night-shift work and occupational fatigue among nurses working 12 h shifts at King Abdulaziz University Hospital (KAUH) in Jeddah, Saudi Arabia. **Methods:** A quantitative, descriptive–correlational, cross-sectional design was used. A convenience sample of 234 registered nurses with more than one year of night-shift experience participated. Data were collected using a structured questionnaire assessing sociodemographic and work-related characteristics; the physiological, psychological, and social impacts of night-shift rotation; and occupational fatigue using the Occupational Fatigue Exhaustion/Recovery (OFER) Scale. **Results:** Most nurses experienced physiological (82.1%), psychological (66.2%), and social (73.9%) impacts of night-shift work. Mean OFER scores were 49.6 ± 28.1 for chronic fatigue, 54.9 ± 16.2 for acute fatigue, and 49.2 ± 12.3 for intershift recovery. Night-shift impacts were positively associated with chronic and acute fatigue and negatively associated with intershift recovery. Social and psychological impacts were independently associated with chronic fatigue and intershift recovery, while social impact and the number of night shifts were independently associated with acute fatigue. **Conclusions:** Twelve-hour night-shift work was associated with substantial physiological, psychological, and social impacts and with higher occupational fatigue and lower intershift recovery. Organizational strategies that limit excessive night duties, protect recovery time, and strengthen staff support may improve nurses’ well-being and performance.

## 1. Introduction

Night-shift work is essential to maintaining continuous hospital care, yet it can disrupt circadian rhythms, sleep, and cognitive functioning. The International Agency for Research on Cancer has defined night-shift work as work involving at least three hours between 23:00 and 06:00 [1]. For nurses, the demands of night work are compounded by prolonged physical, emotional, and cognitive workload, particularly when shifts extend to 12 h [2,3].

Night-shift work has been associated with anxiety, depression, impaired cognition, and cardiometabolic disorders, with potential consequences for both nurses’ health and job performance [4,5]. Occupational fatigue is similarly multidimensional, involving physical, mental, emotional, and social components that may accumulate when work demands exceed opportunities for recovery [6,7]. In nursing, fatigue is particularly important because it can affect concentration, performance, and patient safety [8,9].

The Saudi healthcare system increasingly emphasizes workforce well-being and safer working environments. Previous Saudi research has reported substantial physiological impacts of night-shift rotation and high levels of occupational fatigue among nurses [7,10]. However, evidence focusing specifically on nurses working 12 h night shifts and simultaneously examining physiological, psychological, and social impacts, chronic and acute fatigue, and intershift recovery remains limited.

The nursing workforce in Saudi Arabia and other Gulf Cooperation Council (GCC) countries includes a substantial expatriate workforce, creating a diverse occupational and sociocultural context in which nurses may experience different work-related and personal demands [11,12]. Previous research in Saudi Arabia has identified differences between Saudi and expatriate nurses in areas such as job satisfaction, family–work balance, remuneration, professional opportunities, and workplace experiences [11]. Studies of expatriate nurses have also highlighted factors such as separation from family, language and communication challenges, workload, teamwork, and cultural adjustment as relevant to occupational well-being [11,13]. More broadly, GCC nursing workforce research has emphasized the importance of workload, organizational support, leadership, cultural integration, and employment conditions in understanding nurses’ work experiences [12]. These factors provide an important context for examining whether perceived night-shift impacts and occupational fatigue differ across demographic groups.

Shift work and non-standard working hours have been extensively studied, with established associations with sleep disruption, fatigue, physical and psychological health, work–family conflict, and social well-being. Working Time Society consensus statements emphasize that these outcomes are shaped not only by shift characteristics but also by individual, family, psychosocial, and organizational factors [14]. Night work and extended working hours may interfere with recovery and family and social participation, while individual differences in shift-work tolerance may be related to factors such as gender, family circumstances, social support, and organizational conditions [15]. Perceived control over work scheduling may also be relevant, as greater schedule control has been associated with more favorable occupational well-being outcomes among nurses [16]. Therefore, although the adverse consequences of shift work are well established internationally, their expression may vary according to workforce composition and sociocultural and organizational context. Within Saudi Arabia, further evidence is needed regarding how perceived physiological, psychological, and social impacts of night-shift work are associated with occupational fatigue and intershift recovery among nurses working 12 h shifts, particularly within a nursing workforce comprising both Saudi and expatriate nurses. Therefore, this study aimed to assess the impact of night-shift work and occupational fatigue among nurses working 12 h shifts at King Abdulaziz University Hospital (KAUH) in Jeddah, Saudi Arabia. It also examined associations between sociodemographic and work-related characteristics, night-shift impacts, chronic and acute fatigue, and intershift recovery.

## 2. Materials and Methods

### 2.1. Study Design

A quantitative, descriptive–correlational, cross-sectional design was used.

### 2.2. Setting and Sampling

The study was conducted at King Abdulaziz University Hospital (KAUH) in Jeddah, Saudi Arabia, an academic medical center providing a broad range of inpatient and emergency services.

Participants were recruited using convenience sampling. Eligible participants were registered nurses of both sexes and all nationalities who had more than one year of night-shift experience and were working 12 h shifts in inpatient departments, including medical, surgical, adult medical and surgical intensive care, pediatric and neonatal intensive care, obstetrics and gynecology, labor and delivery, and emergency units. Nursing students, trainees, physicians, technicians, and other allied health professionals were excluded.

The required sample size was calculated using the online Raosoft sample-size calculator (Raosoft, Inc., Seattle, WA, USA) based on a population of 1250 nurses, a 95% confidence level, a 5% margin of error, and a 75% response distribution. After finite-population adjustment, the minimum required sample size was 234 participants. No additional design-effect or nonresponse adjustment was applied. A total of 279 eligible nurses were approached, of whom 41 declined to participate. A total of 238 questionnaires were returned, corresponding to a participation rate of 85.3%. Four questionnaires were excluded because more than 20% of the data were missing, resulting in a final analytical sample of 234 participants (83.9% of those approached).

### 2.3. Data Collection

Data were collected using an anonymous, self-administered English-language questionnaire comprising 40 items in three parts. Part I assessed sociodemographic and work-related characteristics. Part II assessed the impact of night-shift rotations using the 15-item instrument developed by Alsharari [17] and Alsharari et al. [10], with five items each addressing physiological, psychological, and social impacts. The instrument comprises three domains—physiological, psychological, and social impacts—with five items in each domain. Items are positively worded and rated on a four-point Likert scale from strongly agree (1) to strongly disagree (4). Accordingly, higher domain scores indicate greater adverse impact of night-shift work because they reflect greater disagreement with positively framed statements regarding adaptation, well-being, and functioning during night shifts. Each domain has a possible score ranging from 5 to 20. In the original study protocol, domain scores > 10 were classified as indicating impact, whereas scores ≤ 10 were classified as no impact [10,17]. Part III used the 15-item Occupational Fatigue Exhaustion/Recovery (OFER) Scale, which comprises chronic fatigue, acute fatigue, and intershift recovery subscales [18]. The OFER subscales were calculated as the sum of the five items comprising each subscale, divided by 30 and multiplied by 100, producing continuous scores ranging from 0 to 100. Reverse scoring was applied to items 9, 10, 11, 13, and 15. Chronic fatigue comprised items 1–5, acute fatigue items 6–10, and intershift recovery items 11–15. Higher scores indicate a greater level of the respective construct. For comparative purposes, the categories “low,” “low/moderate,” “moderate/high,” and “high” were derived using the quartile-based approach described in the OFER scoring guidance. These categories were used for descriptive comparison and were not interpreted as clinically validated cut-offs or prevalence categories.

Content validity was assessed through expert review by three nursing faculty members holding doctoral degrees and two senior nursing specialists, who evaluated the clarity, relevance, and appropriateness of the questionnaire items. Their feedback resulted in minor modifications to improve clarity and alignment with the study objectives. No quantitative content validity index was prospectively calculated; therefore, no numerical content-validity coefficient is reported. The original authors reported Cronbach’s α coefficients of 0.82 for the Impact of Night-Shift Rotations Scale and 0.85 for the OFER Scale. In the present pilot study, the overall Cronbach’s α was 0.904; domain coefficients are presented in Table 1.

A pilot study was conducted with 24 nurses (approximately 10% of the final sample). Participants reported that the questionnaire was clear and easy to complete, with an average completion time of six minutes. No changes to the instrument or study procedures were required; therefore, the pilot participants were retained in the final dataset.

Data collection began after ethical and institutional permissions were obtained in March 2024 and continued for four months. Eligible nurses were approached in their departments, the study purpose and requirements were explained, and the electronic questionnaire was distributed through electronic platforms. Participants were instructed not to complete the questionnaire more than once. The survey was anonymous, and participants could contact the researcher for clarification or technical support.

### 2.4. Data Analysis

Data were analyzed using IBM Statistical Package for the Social Sciences (SPSS) Statistics version 28 (IBM Corp., Armonk, NY, USA). Numerical variables were summarized using means and standard deviations or medians and ranges, as appropriate. The Mann–Whitney U test was used to compare two independent groups, and the Kruskal–Wallis test was used for comparisons involving more than two groups. Variables that were statistically significant in univariate analyses were entered into multiple linear regression models to identify independent predictors of the physiological, psychological, and social impact scores.

Multiple linear regression models were constructed for the physiological, psychological, and social impact scores. The independent variables included preference for night shifts, number of night shifts during the previous month, gender, and nationality. Predictors were entered sequentially into the regression models, with preference for night shifts entered first, followed by the number of night shifts during the previous month, gender, and nationality. Regression coefficients were reported as unstandardized coefficients (B), standardized coefficients (β), 95% confidence intervals (CIs) for B, and *p*-values. Model fit was assessed using R^2^, and the overall F-test.

Multicollinearity among the independent variables was assessed using the variance inflation factor (VIF) and tolerance statistics. VIF values < 5 and tolerance values > 0.20 were considered to indicate no substantial multicollinearity. The observed VIF and tolerance values were within these acceptable ranges, indicating no evidence of substantial multicollinearity among the independent variables included in the models. Statistical significance was set at *p* ≤ 0.05.

### 2.5. Ethical Considerations

Ethical approval was obtained from the Ethical Committee of the Faculty of Nursing at King Abdulaziz University and subsequently from the Biomedical Ethics Research Committee at King Abdulaziz University Hospital. Participation was voluntary, and completion of the anonymous questionnaire was treated as implied informed consent. Participants were informed about the study and their rights, including the right to withdraw without negative consequences, and confidentiality and anonymity were maintained throughout data collection.

## 3. Results

### 3.1. Sociodemographic and Work-Related Characteristics

Table 2 shows sociodemographic and work-related characteristics among the studied groups. The study included 234 nurses. The average age of the nurses was 37.3 ± 7.8 years, ranging from 23 to 59 years. Most of the nurses (86.8%) were female. About half of the nurses (49.6%) had bachelor’s degrees, 58.1% had more than ten years of nursing experience, and 12.8% were Saudi. Nearly three-fourths of the nurses (76.9%) were married and 68.4% had children. These sociodemographic variables represent key independent variables that were examined to identify their influence on the study’s dependent outcomes, including acute fatigue, chronic fatigue, and intershift recovery.

Nearly one-third of the nurses (35.5%) worked in the intensive care unit, while others worked in emergency, medical, surgical, pediatric, obstetrics, or labor and delivery units. Work-related factors such as department, shift preference, and number of monthly night shifts also served as independent variables in the analysis, as they may contribute to variations in the dependent fatigue-related measures. Only 20.5% of nurses preferred the night shift, and more than one-third had more than eight night shifts during the last month. These work characteristics were statistically assessed in relation to the dependent variables to determine whether certain groups such as those with heavier night workloads or those who dislike night duty were more likely to report higher levels of acute fatigue, chronic fatigue, or poor recovery between shifts.

### 3.2. Physiological, Psychological, and Social Impacts of Night-Shift Work

Table 3 shows the participants’ level of responses regarding the physiological, psychological, and social impacts of night-shift work. Regarding the physiological impact, less than half of the participants (44.9%) agreed that their cycle rhythm can adapt to the night shift, and only 19.2% agreed that they have time for rest during night-shift work. These findings suggest that a considerable proportion of nurses experience circadian misalignment and insufficient rest opportunities during night duties. More than half of the nurses (56%) agreed that they remain active and alert during the night shift, which may reflect a temporary state of compensatory alertness rather than genuine adaptation.

Regarding the psychological impact, nearly two-thirds (61.1%) agreed that they can maintain a positive attitude and behavior during night-shift work, and 64.5% agreed that they are mentally prepared to make the right decisions during night duties. Despite this, only 26.5% agreed that night-shift nurses receive enough recognition and appreciation compared to day-shift nurses, indicating a perceived lack of organizational support. This discrepancy suggests that nurses may exhibit mental readiness during shifts, but still feel undervalued, which can contribute to long-term emotional strain and burnout.

Regarding the social impact, more than one-third of the nurses (34.2%) agreed that they can maintain social relationships while working the night shift (e.g., family and friends), while 64.5% agreed that their families understand their night schedule and provide support. Additionally, 33.3% agreed that they can manage their routine activities and social life (prayers, sports, meetings, shopping, festivals, etc.). These findings highlight that although many nurses receive family support, maintaining social balance remains challenging for a large proportion of the sample. The relatively low agreement percentages in managing social and routine activities emphasize the significant disruption that night-shift work causes in daily life.

Table 4 shows the physiological, psychological, and social impacts of night-shift work. The average physiological impact score was 13 ± 3.1, with 192 nurses (82.1%) reporting a physiological impact, compared to only 42 nurses (17.9%) who did not. This indicates that more than 4 out of every 5 nurses experienced noticeable physical consequences from night shifts.

The psychological impact score averaged 11.7 ± 3.1, where 155 nurses (66.2%) reported psychological effects, while 79 nurses (33.8%) did not. This means that two-thirds of the sample experienced emotional or mental strain related to night work.

The social impact score was 12.5 ± 3.2, with 173 nurses (73.9%) reporting social impact, compared to 61 nurses (26.1%) who did not. Thus, nearly three-quarters of the nurses experienced disruption in family responsibilities, social life, or daily routines due to night shifts.

Overall, the highest proportion of participants meeting the impact criterion was observed for physiological impact (82.1%), followed by social impact (73.9%) and then psychological impact (66.2%).

### 3.3. Factors Associated with the Physiological Impact of Night-Shift Work

Table 5 shows the relationship between sociodemographic and work-related characteristics and the physiological impact of night-shift work. Female nurses had significantly higher physiological impact scores than male nurses (13.3 ± 2.8 vs. 11.3 ± 3.5, *p* = 0.009). Additionally, Saudi nurses had significantly higher physiological impact scores than non-Saudi nurses (14.1 ± 2.9 vs. 12.8 ± 3.0, *p* = 0.021), indicating a significant association between nationality and physiological impact scores.

A significant association was also observed with night-shift preference: nurses who preferred night shifts had the lowest physiological impact score (10.8 ± 2.8), compared to those who did not prefer night shifts (14.4 ± 3.2), *p* < 0.001. Similarly, nurses who worked more than eight night shifts per month reported a significantly higher physiological impact (14 ± 3.0) than those who worked eight or fewer shifts (12.4 ± 2.8), *p* < 0.001. Nurses who worked more than eight night shifts per month had significantly higher physiological impact scores than those who worked eight or fewer night shifts per month.

On the other hand, no statistically significant relationships were found between physiological impact and age, education level, years of experience, marital status, number of children, or department of work (all *p* > 0.05). Although small numerical variations exist across these groups, they did not reach statistical significance, indicating that these characteristics did not meaningfully influence the physiological outcomes in this sample.

### 3.4. Factors Associated with the Psychological Impact of Night-Shift Work

Table 6 shows the relationship between sociodemographic factors and the psychological impact of the night shift. The psychological impact was significantly higher among female nurses compared to males (12 ± 3 vs. 10 ± 3.3, *p* = 0.005), indicating a significant association between gender and psychological impact scores. Similarly, Saudi nurses reported higher psychological impact scores than non-Saudi nurses (13 ± 3 vs. 11.5 ± 3.1, *p* = 0.006), indicating a significant association between nationality and psychological impact scores.

Night-shift preference also showed a strong association: nurses who did not prefer the night shift had the highest psychological impact score (13.1 ± 3.3), compared to those who sometimes preferred night shifts (11.8 ± 2.6) and those who preferred night shifts (9.6 ± 2.8), with a statistically significant difference (*p* < 0.001). Additionally, nurses who worked more than eight night shifts per month had a significantly higher psychological impact (12.7 ± 3.4) than those who worked eight or fewer (11.1 ± 2.8), *p* < 0.001. Nurses who worked more than eight night shifts per month had significantly higher psychological impact scores than those who worked eight or fewer night shifts per month.

On the other hand, there were no statistically significant relationships between psychological impact and age, education level, years of nursing experience, marital status, number of children, or department of work (all *p* > 0.05). Although slight variations in mean scores existed among these groups, they were not large enough to indicate meaningful differences.

### 3.5. Factors Associated with the Social Impact of Night-Shift Work

Table 7 shows the relationship between sociodemographic factors and the social impact of the night shift. The social impact was significantly higher among female nurses than males (12.8 ± 3.1 vs. 10.9 ± 3.4, *p* = 0.011), indicating that women experienced greater disruption in family responsibilities, social routines, and daily activities. Similarly, Saudi nurses reported significantly higher social impact scores compared to non-Saudi nurses (14.2 ± 2.7 vs. 12.3 ± 3.2, *p* = 0.001), indicating a significant association between nationality and social impact scores.

Night-shift preference showed a strong association: nurses who did not prefer night shifts had the highest social impact score (13.7 ± 3.1), followed by those who sometimes preferred night shifts (12.6 ± 2.9), while nurses who preferred night shifts had the lowest social impact (10.8 ± 3.2), *p* < 0.001. In addition, nurses who worked more than eight night shifts per month exhibited significantly higher social impact (13.3 ± 3.4) than those who worked eight or fewer (12.1 ± 3.0), *p* = 0.003. Nurses who worked more than eight night shifts per month had significantly higher social impact scores than those who worked eight or fewer night shifts per month (13.3 ± 3.4 vs. 12.1 ± 3.0, *p* = 0.003).

On the other hand, no statistically significant differences were observed in social impact across age groups, education levels, years of experience, marital status, number of children, or department of work (all *p* > 0.05). Although minor variations existed numerically, they were not meaningful enough to indicate real differences.

### 3.6. Factors Associated with Physiological, Psychological, and Social Impacts of Night-Shift Work

Table 8 shows the multivariate analysis of the physiological, psychological, and social impact scores of night-shift work. After controlling for all sociodemographic and work-related variables, four factors remained significantly associated with all three impact domains in the multivariable analyses: night-shift preference, number of night shifts per month, gender, and nationality.

For the physiological impact, not preferring the night shift had the strongest effect (Beta = 0.381, 95% CI: 1.2–2.1, *p* < 0.001), followed by working more than eight night shifts per month (Beta = 0.273, 95% CI: 1.2–2.3, *p* < 0.001). Female gender (Beta = 0.200, *p* < 0.001) and Saudi nationality (Beta = 0.129, *p* = 0.002) were also significantly associated with higher physiological impact scores. These results indicate that both personal preference and workload intensity independently contribute to physical fatigue, even after adjusting for age, experience, and department.

For the psychological impact, the number of monthly night shifts was significantly associated with psychological impact scores (Beta = 0.366, 95% CI: 0.990–2.4, *p* < 0.001). Nationality (Beta = 0.582, *p* = 0.008), gender (Beta = 0.521, *p* = 0.001), and night-shift preference (Beta = 0.255, *p* < 0.001) were also significantly associated with psychological impact scores.

For the social impact, night-shift preference was significantly associated with social impact scores (Beta = 0.280, 95% CI: 0.6–2.2, *p* < 0.001), as was the number of night shifts during the previous month (Beta = 0.211, *p* < 0.001). Gender (Beta = 0.198, *p* = 0.001) and nationality (Beta = 0.191, *p* = 0.011) were also significantly associated with social impact scores.

Overall, night-shift preference, number of night shifts during the previous month, gender, and nationality remained significantly associated with physiological, psychological, and social impact scores in the multivariable analyses.

### 3.7. Levels of Occupational Fatigue and Intershift Recovery

Table 9 shows the participants’ level of agreement with the Occupational Fatigue Exhaustion Recovery (OFER) Scale. For the Chronic Fatigue State Scale, a noticeable proportion of nurses reported persistent exhaustion: 5.6% strongly agreed that they felt “at the end of their rope,” while 12.8% strongly agreed that they were “living to work,” indicating a sense of overwhelming workload. Additionally, 10.7% strongly agreed that too much was expected of them at work, reflecting perceived work overload.

For the Acute Fatigue State Scale, acute exhaustion after completing shifts was evident, as 17.5% strongly agreed that they felt exhausted upon arriving home, and 15% strongly agreed that they had little energy left after work. In contrast, only 7.7% strongly agreed that they had lots of energy for family or friends, and 8.5% strongly agreed that they had enough energy for hobbies, indicating limited immediate recovery after night duties.

For the Intershift Recovery Scale, 7.7% strongly agreed that they did not have enough time to fully recover after the previous day’s work, while 12.8% strongly agreed that they still felt fatigued from yesterday’s shift when starting work today. Furthermore, only 8.1% strongly agreed that they were fully refreshed by the start of a new shift, highlighting insufficient restorative rest between shifts for many nurses.

Overall, the response patterns reveal that nurses experience both acute and chronic fatigue, with limited intershift recovery, emphasizing the cumulative burden of night-shift work on physical and mental well-being.

Table 10 presents the continuous OFER scores and the descriptive categorical distribution of chronic fatigue, acute fatigue, and intershift recovery. The mean chronic fatigue score was 49.6 ± 28.1, with a median of 50.0 (range: 0–100). The mean acute fatigue score was 54.9 ± 16.2, with a median of 53.3 (range: 0–100), while the mean intershift recovery score was 49.2 ± 12.3, with a median of 50.0 (range: 0–100).

For descriptive comparison, participants were also distributed across the low, low/moderate, moderate/high, and high categories according to the quartile-based approach described in the OFER scoring guidance. These categories were used for comparative descriptive purposes and should not be interpreted as clinically validated cut-offs or prevalence categories. For chronic fatigue, 26.5% of participants were classified in the low category, 21.8% in the low/moderate category, 26.5% in the moderate/high category, and 25.2% in the high category. For acute fatigue, 2.6% were classified as low, 31.2% as low/moderate, 53.8% as moderate/high, and 12.4% as high. For intershift recovery, 3.0% were classified as low, 42.7% as low/moderate, 52.1% as moderate/high, and 2.1% fell within the highest descriptive category.

### 3.8. Factors Associated with Chronic Fatigue

Table 11 shows the relationship between the Chronic Fatigue State Scale and sociodemographic and work-related characteristics. Nurses with more than ten years of experience reported significantly lower chronic fatigue scores compared with those having fewer years of experience (45.4 ± 27.6 vs. 53.7 ± 29.8 for 1–5 years and 56.7 ± 26.8 for 6–10 years, *p* = 0.025). Saudi nurses demonstrated significantly higher chronic fatigue levels than non-Saudi nurses (60.7 ± 25.2 vs. 48 ± 28.2, *p* = 0.020), indicating a notable difference between nationality groups.

Night-shift preference showed a strong association with chronic fatigue: nurses who preferred night duty had the lowest mean fatigue score (39 ± 29.4), compared with 48.3 ± 25.1 among those who “sometimes” preferred night shifts, and 59.8 ± 29.6 among those who did not prefer night shifts (*p* < 0.001). Similarly, nurses who worked ≤8 night shifts per month had significantly lower fatigue scores than those working >8 shifts (46.3 ± 28.0 vs. 55.4 ± 27.7, *p* = 0.017).

No statistically significant differences were found based on age groups (*p* = 0.101), gender (*p* = 0.316), education level (*p* = 0.666), marital status (*p* = 0.209), number of children (*p* = 0.724), or department of work (*p* = 0.317).

### 3.9. Factors Associated with Acute Fatigue

Table 12 shows the relationship between the Acute Fatigue State Scale and sociodemographic and work-related characteristics. Saudi nurses reported significantly higher acute fatigue scores compared with non-Saudi nurses (60.2 ± 15.6 vs. 54.2 ± 16.1, *p* = 0.043). Night-shift preference also showed a strong association with acute fatigue levels: nurses who preferred night duty had the lowest mean score (48.5 ± 16.9), compared with 55.8 ± 16.2 among those who “sometimes” preferred night shifts, and 58.1 ± 14.4 among those who did not prefer night shifts (*p* = 0.005).

Night-shift frequency was another significant factor. Nurses who worked >8 night shifts per month had substantially higher acute fatigue levels than those who worked ≤8 shifts (59.3 ± 16.5 vs. 52.4 ± 15.4, *p* = 0.003).

No statistically significant differences were found based on age groups (*p* = 0.703), gender (*p* = 0.193), education level (*p* = 0.243), nursing experience (*p* = 0.240), marital status (*p* = 0.584), number of children (*p* = 0.180), or department of work (*p* = 0.359).

### 3.10. Factors Associated with Intershift Recovery

Table 13 shows the relationship between the Intershift Recovery State Scale and sociodemographic and work-related characteristics. Female nurses reported significantly lower intershift recovery scores compared with males (48.5 ± 12.4 vs. 53.3 ± 10.7, *p* = 0.008). Nationality also showed a significant association, with Saudi nurses reporting lower recovery levels than non-Saudi nurses (44 ± 12.7 vs. 49.9 ± 12.0, *p* = 0.014).

Night-shift frequency was another significant factor: nurses who worked >8 night shifts per month had lower recovery scores compared with those who worked ≤8 shifts (46.4 ± 11.9 vs. 50.8 ± 12.2, *p* = 0.022).

There were no statistically significant differences in intershift recovery scores based on age groups (*p* = 0.604), education level (*p* = 0.425), years of nursing experience (*p* = 0.142), marital status (*p* = 0.985), presence or number of children (*p* = 0.866, 0.135), department of work (*p* = 0.285), or preference for night shifts (*p* = 0.106).

### 3.11. Associations Between Night-Shift Impacts, Occupational Fatigue, and Intershift Recovery

Table 14 shows the correlation between the Occupational Fatigue Exhaustion Recovery (OFER) subscales and the night-shift impact subscales. There was a significant positive correlation between the physiological night-shift impact score and both chronic fatigue (r = 0.454, *p* < 0.001) and acute fatigue (r = 0.412, *p* < 0.001). A significant negative correlation was found between the physiological impact score and intershift recovery (r = –0.355, *p* < 0.001).

Similarly, the psychological night-shift impact score demonstrated a significant positive correlation with chronic fatigue (r = 0.495, *p* < 0.001) and acute fatigue (r = 0.375, *p* < 0.001), and a significant negative correlation with intershift recovery (r = –0.319, *p* < 0.001).

The social night-shift impact score also showed a significant positive correlation with chronic fatigue (r = 0.577, *p* < 0.001) and acute fatigue (r = 0.433, *p* < 0.001), while showing a significant negative correlation with intershift recovery (r = –0.355, *p* < 0.001).

**Figure 1:**
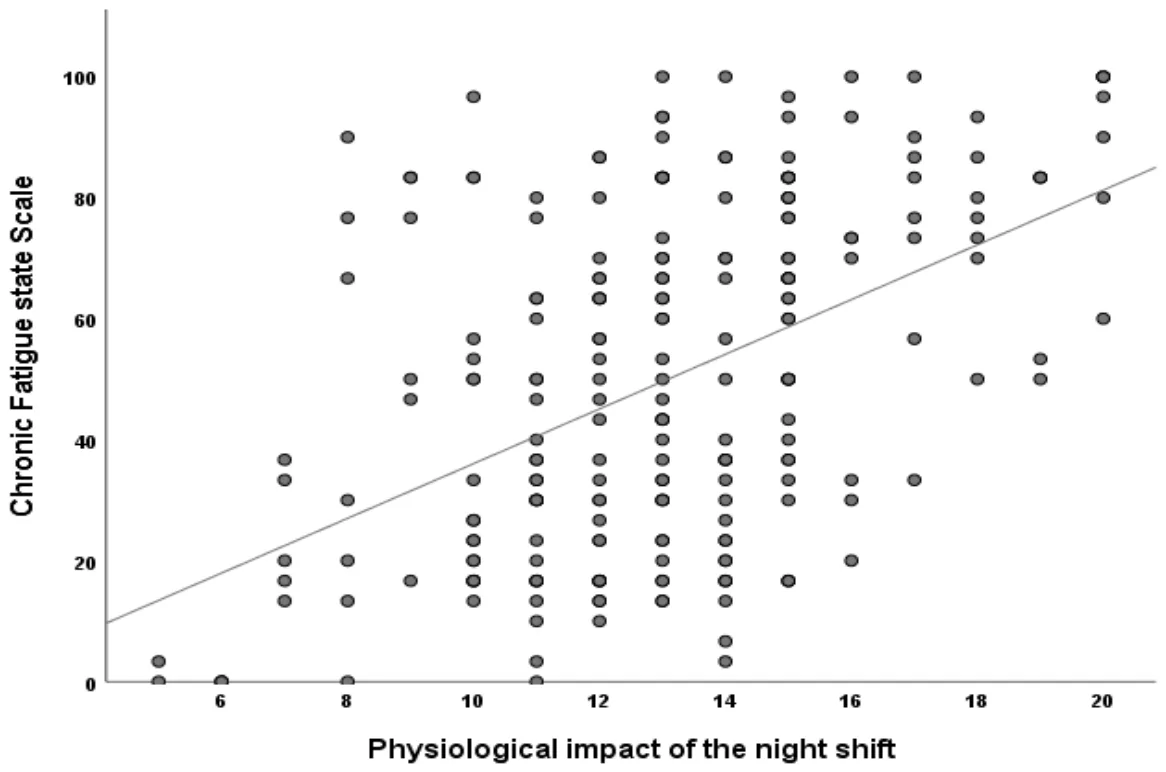
Correlation between chronic fatigue scale and physiological impact of night shifts. Dots represent individual participants’ paired scores, and the solid line represents the fitted linear regression line.

**Figure 2:**
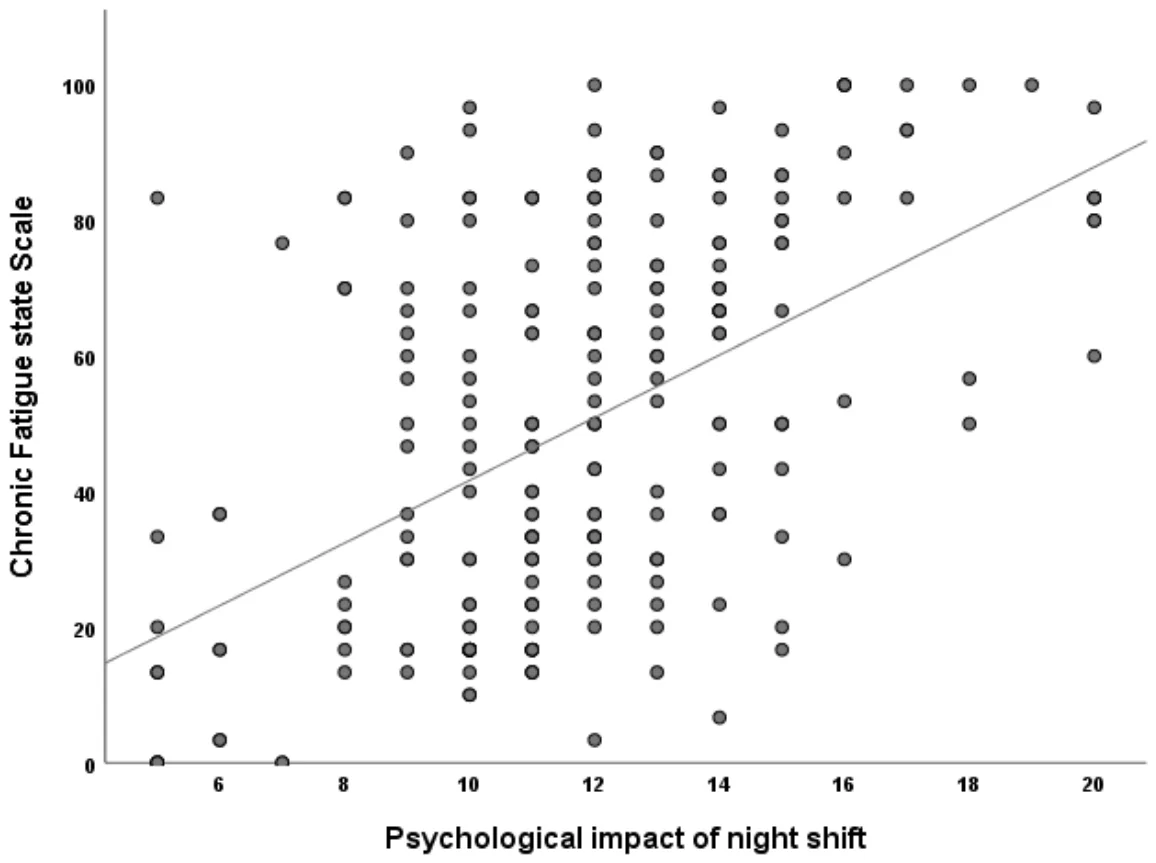
Correlation between chronic fatigue scale and psychological impact of night shifts. Dots represent individual participants’ paired scores, and the solid line represents the fitted linear regression line.

**Figure 3:**
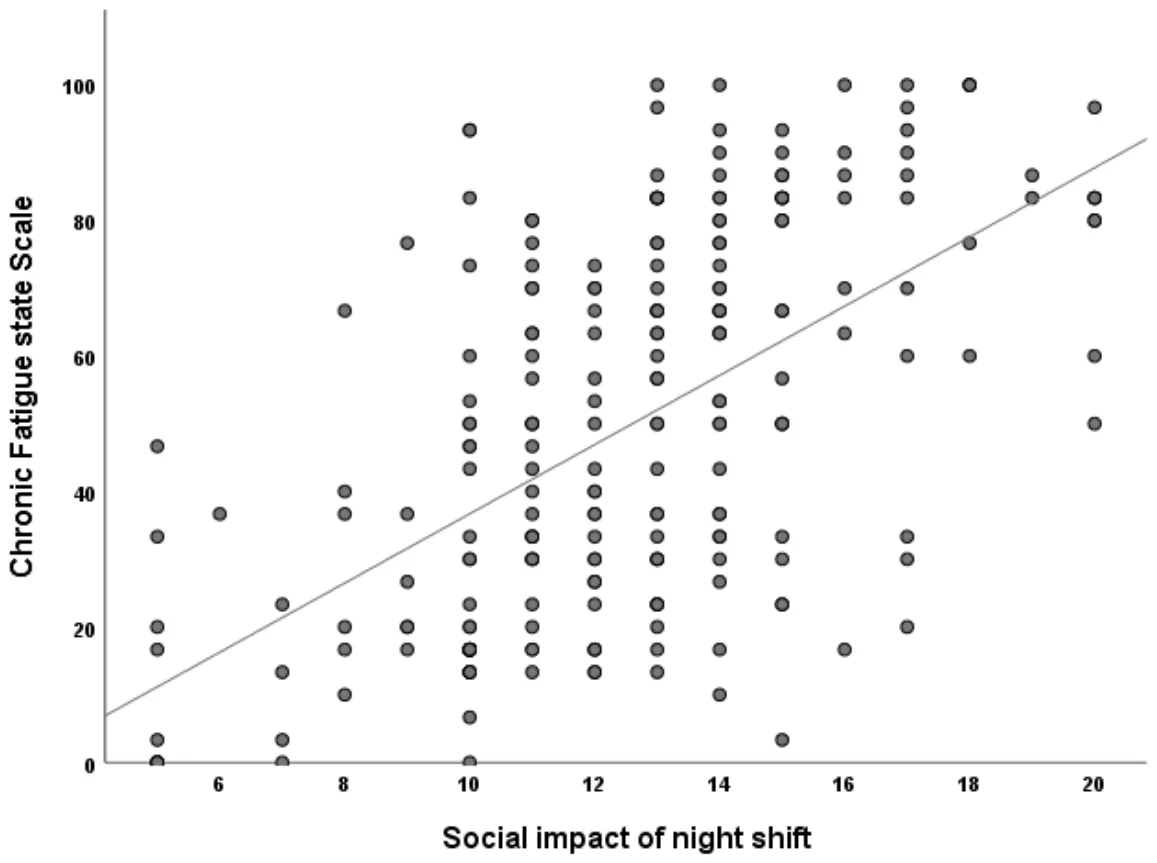
Correlation between chronic fatigue scale and social impact of night shifts. Dots represent individual participants’ paired scores, and the solid line represents the fitted linear regression line.

As shown in Figure 1, chronic fatigue increases as the physiological impact of night-shift work increases, which is consistent with the moderate positive correlation reported in Table 14 (r = 0.454, *p* < 0.001). Similarly, Figure 2 illustrates a positive association between chronic fatigue and psychological impact, supporting the statistically significant correlation (r = 0.495, *p* < 0.001). In addition, Figure 3 shows a strong positive relationship between chronic fatigue and social impact, reflecting the highest correlation observed (r = 0.577, *p* < 0.001).

**Figure 4:**
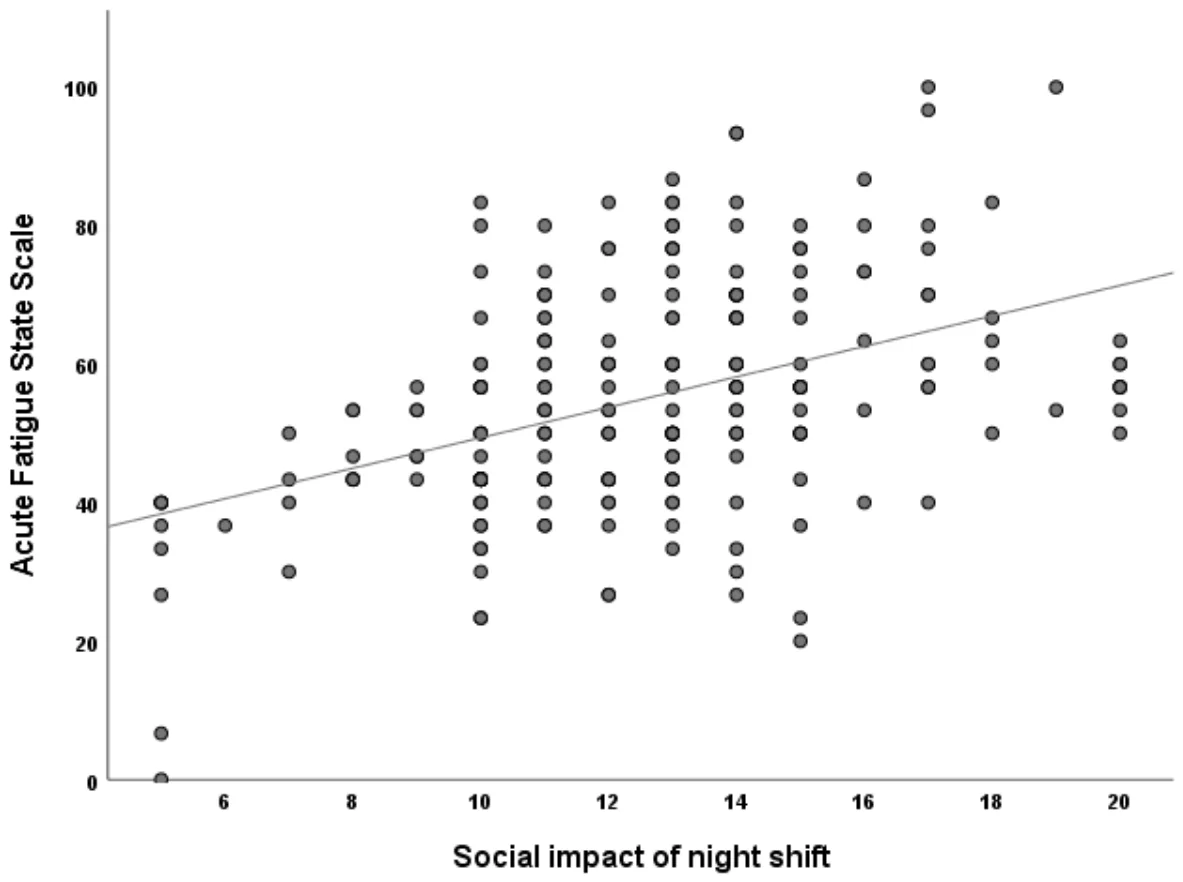
Correlation between Acute Fatigue State Scale and social impact of night shifts. Dots represent individual participants’ paired scores, and the solid line represents the fitted linear regression line.

**Figure 5:**
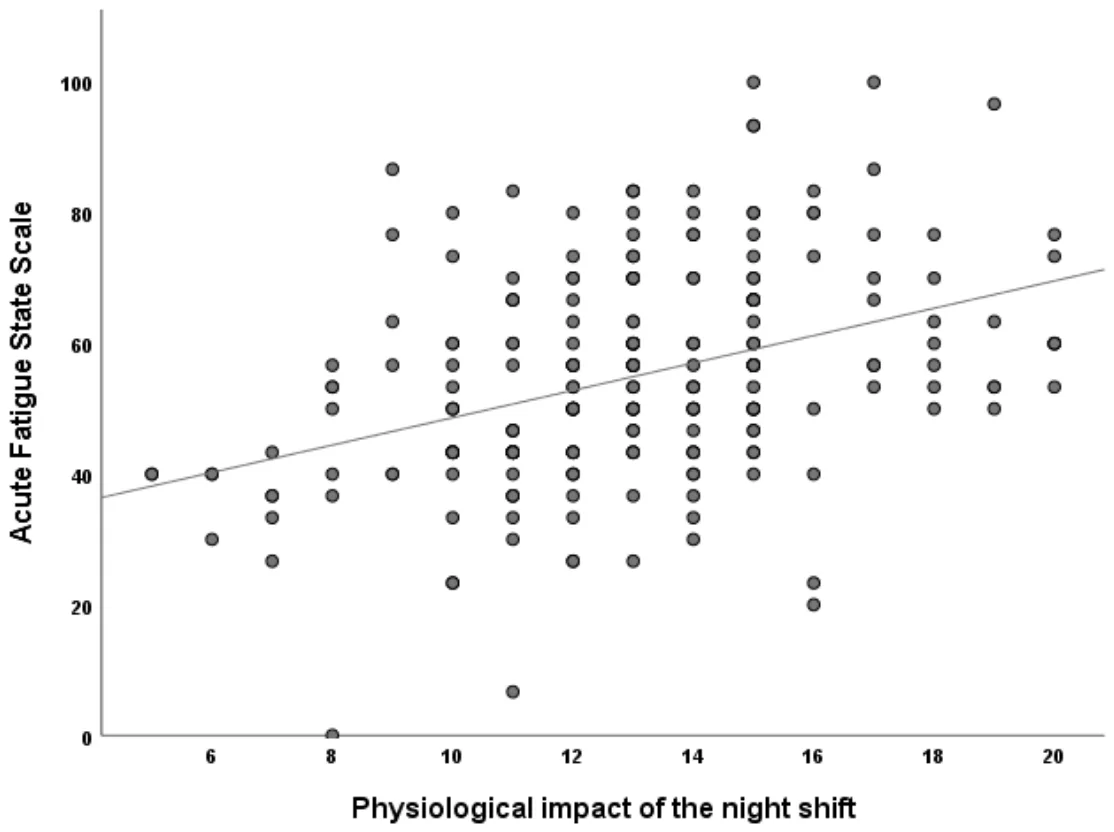
Correlation between Acute Fatigue State Scale and physiological impact of night shifts. Dots represent individual participants’ paired scores, and the solid line represents the fitted linear regression line.

Regarding acute fatigue, Figure 4 demonstrates that higher social impact scores are associated with higher acute fatigue levels, in line with the significant positive correlation reported in Table 14 (r = 0.433, *p* < 0.001). Likewise, Figure 5 shows a positive relationship between physiological impact and acute fatigue, confirming the correlation found in the table (r = 0.412, *p* < 0.001).

### 3.12. Factors Associated with Occupational Fatigue and Intershift Recovery

Table 15 presents the multivariable analysis examining factors associated with chronic fatigue, acute fatigue, and intershift recovery. For the Chronic Fatigue State Scale, the social impact of night shifts was independently associated with chronic fatigue (β = 0.438, 95% CI: 2.5–5.2, *p* < 0.001), as was the psychological impact of night shifts (β = 0.189, 95% CI: 0.3–3.1, *p* = 0.017).

For the Acute Fatigue State Scale, the social impact of night shifts was independently associated with acute fatigue (β = 0.408, 95% CI: 1.5–2.7, *p* < 0.001), as was the number of night shifts during the last month (β = 0.128, 95% CI: 0.4–8.2, *p* = 0.034).

For the Intershift Recovery Scale, both the social impact of night shifts (β = –0.240, 95% CI: –1.6 to –0.295, *p* = 0.004) and the psychological impact of night shifts (β = –0.223, 95% CI: –1.6 to –0.242, *p* = 0.008) were independently associated with intershift recovery.

## 4. Discussion

The present study examined the physiological, psychological, and social impacts of night-shift work and their associations with occupational fatigue and intershift recovery among nurses working 12 h shifts. The findings demonstrate that night-shift work was associated with considerable impact across all three domains. Physiological impacts were the most frequently reported (82.1%), followed by social (73.9%) and psychological (66.2%) impacts. In addition, chronic and acute fatigue were common, while high intershift recovery was uncommon. These findings emphasize that the effects of night work extend beyond physical tiredness and involve interconnected physiological, psychological, and social consequences.

Physiological impacts were particularly prominent. Most nurses reported physiological challenges associated with night work, including disturbances in sleep and reduced alertness. Only a small proportion reported obtaining adequate sleep after night duty, suggesting that the recovery period following night shifts may not sufficiently compensate for the disruption caused by working during normal sleeping hours. Similar findings have been reported internationally, where night and extended shifts have been associated with greater fatigue, impaired alertness, circadian disruption, and insufficient recovery [3,19,20,21,22,23]. Previous research has also demonstrated that fatigue increases with greater night-shift exposure and extended working hours [24,25,26]. In the present study, female gender, Saudi nationality, and working more than eight night shifts per month were associated with higher physiological impact scores, indicating differences in reported physiological impact according to individual and work-related characteristics.

Psychological impacts were also substantial, with approximately two-thirds of participants. Nurses reported emotional exhaustion, irritability, reduced morale, and feelings that their work was insufficiently appreciated. Although many participants reported being able to maintain sufficient concentration for clinical decision-making, the presence of considerable psychological impact remains important because persistent night work may affect emotional well-being even when nurses perceive their immediate cognitive functioning as adequate. Previous research has similarly linked night-shift work with poorer psychological well-being, including increased symptoms of depression, anxiety, stress, and emotional exhaustion [2,27,28]. The greater psychological impact observed among nurses working more frequent night shifts also suggests that reducing excessive exposure to night duty may be important for protecting nurses’ psychological well-being.

Shift preference may also be considered within the broader context of perceived control over work schedules. Previous research among nurses suggests that perceived control over work scheduling and organizational support for scheduling are relevant to nurses’ occupational well-being and work-related outcomes [16,29]. However, the present study assessed nurses’ preference for night-shift work rather than locus of control or actual control over shift allocation. Therefore, the association between shift preference and night-shift impact should not be interpreted as evidence of a locus-of-control effect. Future studies should directly assess perceived schedule control, autonomy over shift allocation, and locus of control to determine whether these factors are associated with nurses’ adaptation to night-shift work. The social impact of night work was likewise considerable, affecting 73.9% of participants. Night shifts can interfere with family responsibilities, social relationships, leisure activities, and participation in normal daytime routines. These effects are particularly important because adequate recovery is not limited to physical rest; opportunities for psychological detachment and social interaction may also contribute to recovery between shifts. The present findings therefore suggest that the consequences of night work should be viewed within a broader occupational and social context rather than solely as a sleep-related problem.

The gender-related differences observed in the present study should also be interpreted within the broader context of work and family responsibilities. Recent research among shift-working nurses has shown that work–family conflict is associated with greater physical and mental fatigue, and that sleep duration and shift-scheduling characteristics may also be relevant to fatigue among nurses [30]. Among married female nurses, the number of night shifts, number of children, childcare pressures, family burden, family harmony, and workplace support have also been associated with different patterns of work–family conflict [31]. Research focusing on female shift-working nurses has additionally demonstrated substantial fatigue and disruption of sleep–wake and circadian patterns, including among nurses working 12 h shifts [21]. These findings provide possible context for the higher physiological, psychological, and social impact scores observed among women in the present study. However, family and domestic responsibilities, work–family conflict, and sleep–wake characteristics were not directly measured in the present study; therefore, these factors should be considered possible contextual explanations rather than established mechanisms underlying the observed gender differences.

The observed differences between Saudi and non-Saudi nurses should be interpreted cautiously. Although nationality was associated with several night-shift impact and fatigue-related outcomes in the present study, nationality itself should not be considered a causal explanation for these differences. Previous research suggests that Saudi and expatriate nurses may differ in several aspects of their occupational and social circumstances, including family responsibilities, social support, living arrangements, migration-related experiences, cultural adjustment, employment conditions, workload distribution, job security, and access to organizational support [11,13]. Within Saudi Arabia and the broader Gulf Cooperation Council (GCC) context, the nursing workforce includes a substantial expatriate component, and migrant nurses may experience distinct occupational and sociocultural circumstances [11,12]. Therefore, the differences observed between Saudi and non-Saudi nurses in this study may reflect a complex combination of sociocultural, migration-related, occupational, and employment factors rather than nationality itself. However, these potential explanatory factors were not directly measured in the present study and should therefore be regarded as possible contextual explanations rather than established causal mechanisms. Furthermore, given the cross-sectional design, the mechanisms underlying the observed nationality-related differences cannot be established. The unequal distribution of Saudi and non-Saudi nurses in the sample (30 versus 204) also warrants caution when interpreting these findings. Future studies with larger and more balanced nationality groups should directly examine the sociocultural, occupational, and organizational factors that may contribute to these differences. Occupational fatigue findings further reinforce this concern. The mean chronic fatigue score was 49.6 ± 28.1, while the mean acute fatigue score was 54.9 ± 16.2. Previous studies have similarly demonstrated substantial chronic and acute fatigue among nurses working night or rotating shifts, particularly when night duties are frequent, shifts are prolonged, overtime is common, or adequate breaks and days off are limited [3,7,24,26]. Acute fatigue may directly reflect the demands of recent work periods, whereas persistent exposure to inadequate recovery may contribute to the development of chronic fatigue. Together, these findings emphasize the importance of considering both immediate and cumulative fatigue when evaluating the effects of night-shift schedules.

The mean intershift recovery score was 49.2 ± 12.3. When the scores were additionally categorized for descriptive comparison, 2.1% of participants fell within the highest categorical level of intershift recovery. Reduced intershift recovery may indicate that nurses begin subsequent shifts without fully restoring their physical and psychological resources. Previous studies have similarly reported reduced intershift recovery among nurses working rotating or night shifts, especially among those experiencing long working hours, frequent night duties, inadequate sleep, or insufficient time off [3,24,26]. Although Alshammari et al. [7] reported comparatively better recovery among Saudi emergency nurses, recovery was still influenced by factors such as family responsibilities and gender, demonstrating that recovery may vary according to both organizational and individual circumstances.

Importantly, the current study found that greater physiological, psychological, and social impacts of night-shift work were associated with greater occupational fatigue and poorer intershift recovery. In the multivariable analyses, social and psychological impacts were independently associated with chronic fatigue and intershift recovery, while social impact and a greater number of night shifts were independently associated with higher acute fatigue. These findings suggest that occupational fatigue among night-shift nurses is multidimensional and cannot be addressed solely by focusing on sleep duration. Scheduling practices, psychological well-being, social demands, and opportunities for meaningful recovery should all be considered when developing strategies to reduce fatigue among nurses working 12 h night shifts. The associations between perceived night-shift impacts, occupational fatigue, and intershift recovery should be interpreted cautiously. Both the night-shift impact domains and the fatigue and recovery outcomes were assessed contemporaneously using self-reported questionnaires. Therefore, the observed associations may partly reflect common-method variance. In addition, some aspects of the psychological and social impact domains may conceptually overlap with fatigue and recovery, which could contribute to the observed associations.

### 4.1. Strengths and Limitations

This study has several strengths. It examined the physiological, psychological, and social impacts of night-shift work together with chronic fatigue, acute fatigue, and intershift recovery among nurses working 12 h shifts. The inclusion of 234 registered nurses provided an adequate sample for examining associations among the study variables, while the use of established instruments and multivariable analyses strengthened the assessment of factors associated with fatigue and recovery.

Nevertheless, several limitations should be considered. The cross-sectional design does not allow causal relationships to be established between night-shift work, occupational fatigue, and recovery. In addition, this study was conducted at a single academic medical center, and participants were recruited using convenience sampling. Consequently, the sample may not fully represent nurses working in other hospitals, healthcare sectors, or geographic regions in Saudi Arabia, and selection bias cannot be excluded. The findings should therefore be interpreted within the context of the participating nurses rather than generalized to the broader Saudi nursing workforce. Future multicenter studies involving nurses from different hospital types, healthcare sectors, and geographic regions are recommended to improve representativeness and the generalizability of the findings.

Reliance on contemporaneous self-reported questionnaire data may introduce recall and social desirability biases, particularly for fatigue, psychological effects, sleep-related experiences, and perceptions of night-shift work. Because both the night-shift impact domains and fatigue and recovery outcomes were assessed using self-report measures at the same time, the observed associations may also be partly influenced by common-method variance. In addition, some aspects of the psychological and social impact domains may conceptually overlap with fatigue and recovery, potentially contributing to the observed associations. The absence of objective measures of sleep and fatigue also prevents independent verification of participants’ reported experiences.

Other factors that could be associated with fatigue and recovery, including sleep duration and quality, workload, family and domestic responsibilities, commuting time, detailed shift characteristics (including permanent versus rotating schedules, consecutive shifts, days on and off, and overtime), individual health characteristics, and organizational factors, were not fully examined, and residual confounding by unmeasured factors cannot be excluded. In addition, objective organizational and performance outcomes, such as sickness absence, occupational injuries, staff turnover, and other human-resources indicators, were not available and therefore could not be examined in relation to night-shift work. Furthermore, the unequal distribution of Saudi and non-Saudi participants (12.8% and 87.2%, respectively) warrants caution when interpreting nationality-related differences. These differences may reflect unmeasured variations in migration-related circumstances, employment conditions, scheduling, occupational roles, family context, living arrangements, social support, or access to institutional support rather than nationality itself.

Another limitation is that baseline psychological and physiological workload was not directly quantified using a validated workload instrument or objective measure. Although nursing department/unit was recorded and analyzed as a work-related characteristic, differences in patient acuity, physical demands, workload intensity, and exposure to emotionally stressful clinical situations may exist between units. These factors may be associated with fatigue and recovery independently of night-shift exposure and may therefore represent potential sources of residual confounding. Future studies should incorporate standardized workload and patient-acuity measures to better account for these differences.

### 4.2. Implications for Practice and Future Research

The findings have important implications for nursing management and occupational health practice. Given the substantial physiological, psychological, and social impacts observed among nurses working 12 h night shifts, healthcare organizations should adopt structured fatigue-management strategies rather than relying solely on individual nurses to manage fatigue. Nursing administrators should review scheduling practices to minimize excessive or consecutive 12 h night shifts and ensure adequate opportunities for intershift recovery, particularly because nurses working more than eight night shifts per month in the present study reported greater night-shift impacts and poorer fatigue-related outcomes. Occupational health guidance recommends minimizing consecutive night shifts and allowing sufficient recovery following extended shifts [32]. Hospitals should also regularly review staffing levels and workload distribution during night shifts and consider flexible scheduling arrangements that account for individual differences in tolerance and recovery needs [15,16].

Structured rest breaks should be incorporated into night-shift scheduling, with adequate staff coverage to enable nurses to take these breaks. Appropriate rest facilities that provide a quiet and comfortable environment should also be considered for night-shift staff [32]. In addition, healthcare organizations should implement fatigue-risk management approaches that include periodic assessment of fatigue, sleep, recovery, and fatigue-related safety concerns among nurses working extended night shifts. Access to occupational health services, psychological support, sleep-health and fatigue-management education, and nonpunitive mechanisms for reporting excessive fatigue should form part of this organizational approach [32]. These measures are particularly relevant given the substantial psychological and social impacts identified in the present study and the observed associations between these impacts, occupational fatigue, and intershift recovery. Finally, staffing and workload policies should be reviewed to reduce excessive work demands during extended night shifts and to ensure that adequate recovery is possible between duties [26].

Future research should use longitudinal or prospective, hypothesis-driven designs to clarify the temporal relationships between night-shift exposure, occupational fatigue, and intershift recovery. Multicenter studies involving nurses from different hospitals and regions of Saudi Arabia would improve the generalizability of the findings. Future studies should incorporate detailed characteristics of shift scheduling, including permanent versus rotating schedules, consecutive night shifts, days on and off, overtime, and mechanisms for accommodating shift preferences, and should examine whether associations between night-shift exposure and fatigue differ according to gender and other demographic, family, occupational, and organizational characteristics. Additional factors such as sleep quality and duration, workload, family responsibilities, chronotype, and organizational support, perceived schedule control, autonomy over shift allocation, and locus of control should also be examined. Prospective studies should complement self-reported measures with objective assessments, such as actigraphy, sleep diaries, wearable-device data, or appropriate physiological indicators, to provide a more comprehensive assessment of sleep, fatigue, and recovery. Intervention studies are particularly needed to determine whether modifications to shift scheduling, protected recovery periods, and fatigue-management programs can improve nurses’ recovery, well-being, and work-related outcomes.

## 5. Conclusions

Among nurses working 12 h shifts at King Abdulaziz University Hospital, night-shift work was associated with substantial physiological, psychological, and social impacts and with higher occupational fatigue and lower intershift recovery. Physiological impact was reported by 82.1% of participants, psychological impact by 66.2%, and social impact by 73.9%. Chronic and acute fatigue were positively related to night-shift impacts, whereas intershift recovery was negatively related. Social and psychological impacts were associated with chronic fatigue and intershift recovery, while social impact and a greater number of night shifts were independently associated with higher acute fatigue. These findings support fatigue-management strategies that combine balanced scheduling, adequate recovery time, supportive work environments, and staff education.

## Figures and Tables

**Table 1:** Internal consistency reliability of the study instruments.

No.	Domain	No. of Items	Cronbach’s Alpha
D1	Impact of Night-Shift Rotations	15.0	0.850
D1.1	Physiological Impact	5.0	0.779
D1.2	Psychological Impact	5.0	0.789
D1.3	Social Impact	5.0	0.887
D2	Occupational Fatigue Exhaustion/Recovery (OFER)	15.0	0.959
Total		30.0	0.904

**Table 2:** Sociodemographic and work-related characteristics among the studied groups.

Characteristics	Total (n = 234)
Age (years)	
Mean ± SD	37.3 ± 7.8
Median (range)	36 (23–59)
Age group (years)	
21–30	46 (19.7%)
31–40	126 (53.8%)
41–50	44 (18.8%)
>50	18 (7.7%)
Gender	
Female	203 (86.8%)
Male	31 (13.2%)
Education level	
Bachelor’s degree	116 (49.6%)
Diploma in Nursing	112 (47.9%)
Master’s degree	6 (2.6%)
Years of nursing experience	
1–5 years	40 (17.1%)
6–10 years	58 (24.8%)
>10 years	136 (58.1%)
Nationality	
Non-Saudi	204 (87.2%)
Saudi	30 (12.8%)
Marital status	
Married	180 (76.9%)
Single	48 (20.5%)
Divorced	5 (2.1%)
Widow	1 (0.4%)
Children	
No	74 (31.6%)
Yes	160 (68.4%)
Number of children (n = 160)	
One child	68 (42.5%)
Two children	66 (41.3%)
Three children	17 (10.6%)
More than three children	9 (5.6%)
Department work	
Emergency room	34 (14.5%)
Intensive care units “MICU, SICU, PICU and NICU”	83 (35.5%)
Labor and delivery	15 (6.4%)
Medical	33 (14.1%)
Obstetrics and gynecology	11 (4.7%)
Pediatric	27 (11.5%)
Surgical	31 (13.2%)
Do You Prefer Night Shifts?	
No	66 (28.2%)
Sometimes	120 (51.3%)
Yes	48 (20.5%)
How many night shifts did you have in the last month?	
Mean ±SD	8.2 ± 3.2
Median (range)	8 (0–27)
Night shifts during last month	
≤8	148 (63.2%)
>8	86 (36.8%)

**Table 3:** Participants’ level of responses with physiological, psychological and social impacts of night-shift work.

Physiological Impact				
	Strongly Agree	Agree	Disagree	Strongly Disagree
My body’s cycle rhythm can adapt to the night shift	25 (10.7%)	105(44.9%)	83 (35.5%)	21 (9%)
I get enough time to sleep after my night shift	18 (7.7%)	98 (41.9%)	85 (36.3%)	33 (14.1%)
I have a healthy lifestyle when working the night shift	14 (6%)	53 (22.6%)	125 (53.4%)	42 (17.9%)
There is time for rest during night-shift work	14 (6%)	45 (19.2%)	106 (45.3%)	69 (29.5%)
I remain active and alert during night shift	39 (16.7%)	131 (56%)	44 (18.8%)	20 (8.5%)
**Psychological Impact**				
I can maintain a positive attitude and behavior during night-shift work	39 (16.7%)	143 (61.1%)	40 (17.1%)	12 (5.1%)
I am satisfied with my night work schedule	30 (12.8%)	98 (41.9%)	82 (35%)	24 (10.3%)
I don’t feel depressed and isolated when working the night shift	25 (10.7%)	123 (52.6%)	58 (24.8%)	28 (12%)
I am mentally prepared so I can make the right decisions during night-shift work	41 (17.5%)	151 (64.5%)	31 (13.2%)	11 (4.7%)
Night shift nurses get enough recognition and appreciation compared to day-shift nurses	17 (7.3%)	62 (26.5%)	116 (49.6%)	39 (16.7%)
**Social Impact**				
I can maintain social relationships when working the night shift (e.g., family and friends)	22 (9.4%)	80 (34.2%)	96 (41%)	36 (15.4%)
I can manage my home and attend to my family’s needs when working the night shift	18 (7.7%)	75 (32.1%)	109 (46.6%)	32 (13.7%)
My family understands my night schedule and supports me	38 (16.2%)	151 (64.5%)	34 (14.5%)	11 (4.7%)
Home–work transportation is handy (easy) when working the night shift	23 (9.8%)	106 (45.3%)	79 (33.8%)	26 (11.1%)
I can manage my routine activities and social life (prayers, sports, meetings, shopping, festivals, etc.)	16 (6.8%)	78 (33.3%)	99 (42.3%)	41 (17.5%)

**Table 4:** Physiological, psychological, and social impacts of night-shift work.

	Total (n = 234)
Physiological impact score	
Mean ± SD	13 ± 3.1
Median (range)	13 (5–20)
Physiological impact	
No (scored ≤ 10)	42 (17.9%)
Yes (scored > 10)	192 (82.1%)
Psychological impact score	
Mean ± SD	11.7 ± 3.1
Median (range)	12 (5–20)
Psychological impact	
No (scored ≤ 10)	79 (33.8%)
Yes (scored > 10)	155 (66.2%)
Social impact score	
Mean ± SD	12.5 ± 3.2
Median (range)	13 (5–20)
Social impact	
No (scored ≤ 10)	61 (26.1%)
Yes (scored > 10)	173 (73.9%)

**Table 5:** Relationship between sociodemographic and work-related characteristics and physiological impact of night shifts.

Characteristics	Physiological Impact Score			95% Confidence Interval	Effect Size
	Mean ± SD	Median (Range)	*p*-Value		
Age group (years)					
21–30	13.1 ± 3.2	13 (5–20)	0.267 *	(0.252–0.269)	0.004
31–40	13.3 ± 2.7	13 (6–20)			
41–50	12.5 ± 3.1	13 (6–20)			
>50	12.1 ± 3.8	11.5 (5–20)			
Gender					
Female	13.3 ± 2.8	13 (6–20)	0.009 **	(0.007–0.010)	0.17
Male	11.3 ± 3.5	12 (5–18)			
Education level					
Bachelor’s degree	12.8 ± 3.3	13 (5–20)	0.497 *	(0.494–0.513)	<0.001
Diploma in Nursing	13.2 ± 2.7	13 (6–20)			
Master’s degree	13.7 ± 3.7	15 (7–17)			
Years of nursing experience					
1–5 years	13.3 ± 3.1	13 (6–20)	0.736 *	(0.731–0.748)	<0.001
6–10 years	13 ± 2.8	13 (5–20)			
>10 years	12.9 ± 3.1	13 (5–20)			
Nationality					
Non-Saudi	12.8 ± 3.0	13 (5–20)	0.021 **	(0.017–0.023)	0.15
Saudi	14.1 ± 2.9	14.5 (7–20)			
Marital status					
Married	13.0 ± 2.9	13 (6–20)	0.493 *	(0.488–0.508)	<0.001
Single	12.6 ± 3.1	13 (5–20)			
Divorced/Widow	14.7 ± 5.1	15 (7–20)			
Children					
No	13 ± 3.1	13 (5–20)	0.767 **	(0.756–0.773)	0.02
Yes	13 ± 3	13 (6–20)			
Number of children					
One child	12.8 ± 3.3	13.0 (6–20)	0.563 *	(0.858–0.875)	<0.001
Two children	13.1 ± 2.5	13 (8–20)			
Three children	13.5 ± 3.0	13 (9–19)			
More than three children	13.1 ± 3.7	13 (7–20)			
Department work					
Emergency room	13.1 ± 3.0	13 (7–20)	0.884 *	(0.879–0.892)	<0.001
Intensive care unit	12.9 ± 2.9	13 (5–20)			
“MICU, SICU, PICU and NICU”					
Labor and delivery	12.9 ± 3.6	13 (8–20)			
Medical	13.0 ± 3.4	13 (5–20)			
Obstetrics and gynecology	13.5 ± 3.3	14 (7–17)			
Pediatric	12.6 ± 2.1	12 (10–18)			
Surgical	13.4 ± 3.2	13 (6–19)			
Do You Prefer Night Shifts?					
No	14.4 ± 3.2	14 (6–20)	<0.001 *	(<0.001–<0.001)	0.184
Sometimes	13.1 ± 2.5	13 (6–19)			
Yes	10.8 ± 2.8	11 (5–20)			
Night shifts during last month					
≤8-night shift days/month	12.4 ± 2.8	13 (5–20)	<0.001 **	(<0.001–<0.001)	0.28
>8-night shift days/month	14 ± 3	14 (7–20)			

* Kruskal–Wallis test, ** Mann–Whitney test.

**Table 6:** Relationship between sociodemographic factors and psychological impact of night shifts.

Characteristics	Psychological Impact of the Night Shift			95% Confidence Interval	Effect Size
	Mean ± SD	Median (Range)	*p*-Value		
Age group (years)					
21–30	11.5 ± 3.2	11 (5–20)	0.781 *	(0.776–0.792)	<0.001
31–40	11.9 ± 3.1	12 (5–20)			
41–50	11.3 ± 2.8	12 (5–18)			
>50	11.8 ± 3.9	11.5 (6–20)			
Gender					
Female	12 ± 3	12 (5–20)	0.005 **	(0.004–0.007)	0.17
Male	10 ± 3.3	10 (5–16)			
Education level					
Bachelor’s degree	11.5 ± 3.2	11 (5–20)	0.671 *	(0.67–0.69)	<0.001
Diploma in Nursing	12 ± 2.9	12 (6–20)			
Master’s degree	11.7 ± 4.5	12 (5–17)			
Years of nursing experience					
1–5 years	12.1 ± 3.2	11 (5–20)	0.667 *	(0.653–0.672)	<0.001
6–10 years	11.7 ± 3.2	11.5 (5–20)			
>10 years	11.6 ± 3.1	12 (5–20)			
Nationality					
Non-Saudi	11.5 ± 3.1	11 (5–20)	0.006 **	(0.004–0.006)	0.18
Saudi	13 ± 3	13.5 (5–20)			
Marital status					
Married	11.7 ± 3.1	12 (5–20)	0.301 *	(0.344–0.362)	<0.001
Single	11.4 ± 3.0	11 (5–18)			
Divorced/Widow	14.2 ± 5.4	15 (6–20)			
Children					
No	11.6 ± 3.3	11.5 (5–20)	0.859 **	(0.853–0.867)	0.02
Yes	11.8 ± 3.1	12 (5–20.0			
Number of children					
One child	11.7 ± 3.5	11 (5–20)	0.663 *	(0.663–0.682)	<0.001
Two children	11.7 ± 2.6	12 (6–20)			
Three children	12 ± 3.5	13 (5–17)			
More than three children	12 ± 3.5	13 (5–17)			
Department work					
Emergency room	12.1 ± 3.3	12 (5–19)	0.467 *	(0.460–0.479)	<0.001
Intensive care unit	11.4 ± 2.7	12 (5–20)			
“MICU, SICU, PICU and NICU”					
Labor and delivery	12.7 ± 4.7	14 (5–20)			
Medical	11.2 ± 3.4	11 (5–20)			
Obstetrics and gynecology	11.5 ± 3.4	12 (6–18)			
Pediatric	11.8 ± 2.3	11 (8–20)			
Surgical	12.4 ± 3.2	12 (5–20)			
Do You Prefer Night Shifts?					
No	13.1 ± 3.3	13 (5–20)		(0.001–0.002)	0.17
Sometimes	11.8 ± 2.6	12 (5–20)	<0.001 *		
Yes	9.6 ± 2.8	10 (5–20)			
Night shifts during last month					
≤8	11.1 ± 2.8	11 (5–20)	<0.001 **	(0.001–0.002)	0.25
>8	12.7 ± 3.4	12 (5–20)			

* Kruskal–Wallis test, ** Mann–Whitney test.

**Table 7:** Relationship between sociodemographic factors and social impact of night shifts.

Characteristics	Social Impact of Night Shift			95% Confidence Interval	Effect Size
	Mean ± SD	Median (Range)	*p*-Value		
Age group (years)					
21–30	13 ± 3.4	13 (5–20)	0.128 *	(0.117–0.130)	0.003
31–40	12.8 ± 3.2	13 (5–20)			
41–50	11.7 ± 2.5	12 (5–17)			
>50	12 ± 3.9	11.5 (5–20)			
Gender					
Female	12.8 ± 3.1	13 (5–20)	0.011 **	(0.008–0.012)	0.17
Male	10.9 ± 3.4	11 (5–16)			
Education level					
Bachelor’s degree	12.3 ± 3.5	1 (5–20)	0.415 *	(0.413–0.432)	<0.001
Diploma in Nursing	12.7 ± 2.8	13 (5–20)			
Master’s degree	14.0 ± 3.1	14.5(10–17)			
Years of nursing experience					
1–5 years	13.2 ± 3.4	13 (5–20)	0.208 *	(0.198–0.214)	0.005
6–10 years	12.8 ± 3.4	13 (5–20)			
>10 years	12.2 ± 3.0	12 (5–20)			
Nationality					
Non-Saudi	12.3 ± 3.2	12 (5–20)	0.001 **	(0.001–0.003)	0.21
Saudi	14.2 ± 2.7	14 (10–20)			
Marital status					
Married	12.6 ± 3.1	12.5 (5–20)	0.676 *	(0.678–0.696)	<0.0001
Single	12.3 ± 3.1	12.3 (3.1–19)			
Divorced/widow		15 (5–20)			
Children					
No	12.6 ± 3.3	13 (5–20)	0.614 **	(0.299–0.317)	0.03
Yes	12.5 ± 3.1	12 (5–20)			
Number of children					
One child	12.5 ± 3.8	13 (5–20)	0.709 *	(0.708–0.725)	<0.001
Two children	12.3 ± 2.7	12 (5–20)			
Three children	13.2 ± 2.5	12 (10–17)			
More than three children	12.7 ± 2.2	13 (10–16)			
Department work					
Emergency room	12.5 ± 3.1	13 (5–19)	0.666 *	(0.661–0.679)	<0.001
Intensive care unit	12.4 ± 3.1	12 (5–20)			
“MICU, SICU, PICU and NICU”					
Labor and delivery	13.9 ± 3.8	14 (5–20)			
Medical	12.7 ± 3.5	13 (5–20.0)			
Obstetrics and gynecology	12.2 ± 3.1	12 (6–17)			
Pediatric	12.7 ± 2.9	13 (7–20)			
Surgical	12.1 ± 3.0	11 (5–20)			
Do You Prefer Night Shifts?					
No	13.7 ± 3.1	14 (7–20)			
Sometimes	12.6 ± 2.9	13 (5–20)	<0.001 *	(0.001–0.002)	0.088
Yes	10.8 ± 3.2	11 (5–20)			
Night shifts during last month					
≤8	12.1 ± 3.0	12 (5–20)	0.003 **	(0.001–0.003)	0.2
>8	13.3 ± 3.4	14 (5–20)			

* Kruskal–Wallis test, ** Mann–Whitney test.

**Table 8:** Multivariate analysis of the physiological, psychological, and social impact of night-shift scores.

Physiological Impact Score											
	Standardized Beta Coefficient *	Unstandardized Coefficients	Standard Error	95% Confidence Interval for the Beta Coefficient	*p*-Value	VIF	Tolerance	F	R^2^	R	Regression df
Preference for night shift	0.381	1.7	0.2	1.2–2.1	<0.001	1.012	0.989	20.216	0.261	0.511	4
Number of night shifts during last month	0.273	0.2	0.1	1.2–2.3	<0.001	1.003	0.997				
Gender	0.2	1.7	0.5	0.9–2.7	<0.001	1.008	0.992				
Nationality	0.129	1.1	0.5	0.2–2.1	0.002	1.008	0.994				
**Psychological Impact Score**											
	Beta Coefficient *	**Unstandardized Coefficients**	**Standard Error**	**95% Confidence Interval for the Beta Coefficient**	***p*** **-Value**	**VIF**	**Tolerance**	**F**	**R^2^**	**R**	**Regression df**
Preference for night shift	0.255	1.6	0.2	1.1–2.1	<0.001	1.012	0.989	18.055	0.24	0.491	4
Number of night shifts during last month	0.366	0.2	0.1	1.1–2.1	<0.001	1.003	0.997				
Gender	0.521	1.8	0.5	0.8–2.8	0.001	1.008	0.992				
Nationality	0.582	1.3	0.5	0.4–2.4	0.008	1.006	0.994				
**Social Impact Score**											
	Beta Coefficient *	**Unstandardized Coefficients**	**Standard Error**	**95% Confidence Interval for the Beta Coefficient**	***p*** **-Value**	**VIF**	**Tolerance**	**F**	**R^2^**	**R**	**Regression df**
Preference for night shift	0.28	1.3	0.2	0.6–2.2	<0.001	1.012	0.989	12.422	0.178	0.422	4
Number of night shifts during last month	0.211	0.1	0.06	0.6–2.2	<0.001	1.003	0.997				
Gender	0.198	1.7	0.6	0.8–2.9	0.001	1.008	0.992				
Nationality	0.191	1.8	0.6	0.7–2.9	0.011	1.006	0.994				

* Linear regression.

**Table 9:** Participants’ level of responses for the Occupational Fatigue Exhaustion Recovery Scale.

	Strongly Disagree	Disagree	Slightly Disagree	Neither Agree or Disagree	Slightly Agree	Agree	Strongly Agree
OFER Chronic Fatigue (CF) State Scale							
I felt “at the end of my rope” with my work today	16 (6.8%)	81 (34.6%)	20 (8.5%)	46 (19.7%)	23 (9.8%)	35 (15%)	13 (5.6%)
I dreaded waking up to another day of my work today	17 (7.3%)	65 (27.8%)	23 (9.8%)	40 (17.1%)	23 (9.8%)	42 (17.9%)	24 (10.3%)
I wondered how long I can keep going at my work today	20 (8.5%)	62 (26.5%)	20 (8.5%)	30 (12.8%)	30 (12.8%)	48 (20.5%)	24 (10.3%)
I felt that most of the time I was “living to work” today	21 (9%)	50 (21.4%)	20 (8.5%)	30 (12.8%)	32 (13.7%)	51 (21.8%)	30 (12.8%)
Too much was expected of me in my work today	15 (6.4%)	48 (20.5%)	18 (7.7%)	43 (18.4%)	24 (10.3%)	61 (26.1%)	25 (10.7%)
OFER Acute Fatigue State Scale							
After work I had little energy left today	16 (6.8%)	49 (20.9%)	18 (7.7%)	30 (12.8%)	30 (12.8%)	56 (23.9%)	35 (15%)
I felt exhausted when I got home from work today	24 (10.3%)	43 (18.4%)	11 (4.7%)	27 (11.5%)	34 (14.5%)	54 (23.1%)	41 (17.5%)
My work drained my energy completely today	25 (10.7%)	43 (18.4%)	15 (6.4%)	32 (13.7%)	28 (12%)	61 (26.1%)	30 (12.8%)
I had lots of energy to give to my family or friends today	20 (8.5%)	55 (23.5%)	20 (8.5%)	51 (21.8%)	38 (16.2%)	32 (13.7%)	18 (7.7%)
I had plenty of energy left for my hobbies and other activities after I finished work today	25 (10.7%)	61 (26.1%)	21 (9%)	44 (18.8%)	37 (15.8%)	26 (11.1%)	20 (8.5%)
Intershift Recovery Scale							
I didn’t have enough time to recover my energy completely after working yesterday	21 (9%)	47 (20.1%)	23 (9.8%)	45 (19.2%)	39 (16.7%)	41 (17.5%)	18 (7.7%)
If I was tired from work yesterday, I was fully refreshed by the start of work today	17 (7.3%)	41 (17.5%)	20 (8.5%)	37 (15.8%)	46 (19.7%)	54 (23.1%)	19 (8.1%)
I didn’t recover my strength fully from my work yesterday	16 (6.8%)	47 (20.1%)	22 (9.4%)	46 (19.7%)	32 (13.7%)	52 (22.2%)	19 (8.1%)
Recovering from yesterdays’ work fatigue wasn’t a problem for me today	21 (9%)	56 (23.9%)	30 (12.8%)	38 (16.2%)	37 (15.8%)	32 (13.7%)	20 (8.5%)
I was still feeling fatigued from work yesterday when I started work today	16 (6.8%)	49 (20.9%)	20 (8.5%)	42 (17.9%)	30 (12.8%)	47 (20.1%)	30 (12.8%)

**Table 10:** Chronic fatigue, acute fatigue, and intershift recovery scores on the Occupational Fatigue Exhaustion/Recovery (OFER) Scale.

	Chronic Fatigue State Scale
Mean ± SD	49.6 ± 28.1
Median (range)	50 (0–100%)
Low	62 (26.5%)
Low/moderate	51 (21.8%)
Moderate/high	62 (26.5%)
High	59 (25.2%)
	**Acute Fatigue State Scale**
Mean ± SD	54.9 ± 16.2
Median (range)	53.3 (0–100)
Low	6 (2.6%)
Low/moderate	73 (31.2%)
Moderate/high	126 (53.8%)
High	29 (12.4%)
	**Intershift Recovery Scale**
Mean ± SD	49.2 ± 12.3
Median (range)	50 (0–100)
Low	7 (3%)
Low/moderate	100 (42.7%)
Moderate/high	122 (52.1%)
High	5 (2.1%)

**Table 11:** Relationship between Chronic Fatigue Scale and sociodemographic and work-related characteristics.

Characteristics	Chronic Fatigue State Scale				
	Mean ± SD	Median (Range)	*p*-Value	95% Confidence Interval	Effect Size
Age group (years)					
21–30	56.5 ± 28.5	60 (0–100)	0.101 *	(0.094–0.105)	0.014
31–40	50.2 ± 27.9	50 (0–100)			
41–50	43 ± 28	40 (0–100)			
>50	44.1 ± 27.1	36.7 (3.3–96.7)			
Gender					
Female	50.4 ± 28.2	50 (0–100)	0.316 **	(0.301–0.319)	0.07
Male	44.7 ± 27.9	40 (0–86.7)			
Education level					
Bachelor’s degree	51.1 ± 28.1	50 (0–100)	0.666 *	(0.664–0.682)	<0.001
Diploma in Nursing	48.1 ± 28.1	50 (0–100)			
Master’s degree	48.9 ± 32.5	50 (13.3–93.3)			
Years of nursing experience					
1–5 years	53.7 ± 29.8	53.3 (0–100)	0.025 *	(0.021–0.027)	0.023
6–10 years	56.7 ± 26.8	60 (0–100)			
>10 years	45.4 ± 27.6	40 (0–100)			
Nationality					
Non-Saudi	48 ± 28.2	48.3 (0–100)	0.020 **	(0.017–0.023)	0.21
Saudi	60.7 ± 25.2	65 (13.3–100)			
Marital status					
Married	48.9 ± 28.2	50 (0–100)	0.209 *	(0.199–0.215)	0.005
Single	49.9 ± 26.7	45 (0–100)			
Divorced/Widow	70.6 ± 35.4	83.3 (16.7–100)			
Children					
No	53.7 ± 29.1	58.3 (0–100)	0.120 **	(0.110–0.123)	0.010
Yes	47.7 ± 27.6	48.3 (0–100)			
Number of children					
One child	48.4 ± 28.5	51.7 (0–100)	0.724 *	(0.700–0.718)	<0.001
Two children	47.8 ± 26.3	50 (0–100)			
Three children	49 ± 28.2	33.3 (16.7–93.3)			
More than three children	40 ± 32.2	23.3 (13.3–100)			
Department work					
Emergency room	56.1 ± 27.2	60 (13.3–100)	0.317 *	(0.312–0.330)	0.005
Intensive care unit Includes “MICU, SICU, PICU and NICU”	45.7 ± 27.1	40 (0–100)			
Labor and delivery	60.9 ± 35.1	66.7 (0–100)			
Medical	54.9 ± 32.7	60 (0–100)			
Obstetrics and gynecology	43.9 ± 19.1	36.7 (16.7–76.7)			
Pediatric	48.8 ± 27.8	50 (13.3–90)			
Surgical	44.8 ± 24.5	50 (0–86.7)			
Do You Prefer Night Shifts?					
No	59.8 ± 29.6	66.7 (0–100)	<0.001 *	(0.001–0.002)	0.060
Sometimes	48.3 ± 25.1	50 (0–100)			
Yes	39 ± 29.4	28.3 (0–100)			
Night shifts during last month					
≤8	46.3 ± 28.0	43.3 (0–100)	0.017 **	(0.014–0.019)	0.16
>8	55.4 ± 27.7	56.7 (3.3–100)			

* Kruskal–Wallis test, ** Mann–Whitney test.

**Table 12:** Relationship between Acute Fatigue State Scale and sociodemographic and work-related characteristics.

Characteristics	Acute Fatigue State Scale			95% Confidence Interval	Effect Size
	Mean ± SD	Median (Range)	*p*-Value		
Age group (years)					
21–30	57.7 ± 17.1	56.7 (23.3–100)	0.703 *	(0.697–0.715)	<0.001
31–40	54.4 ± 15.2	53.3 (6.7–93.3)			
41–50	54.4 ± 18.3	51.7 (0–96.7)			
>50	53.1 ± 15.4	51.7 (23.3–93.3)			
Gender					
Female	55.4 ± 16.3	53.3 (0–100)	0.193 **	(0.188–0.204	0.09
Male	51.7 ± 15.4	50 (20–93.3)			
Education level					
Bachelor’s degree	54.6 ± 16.4	53.3 (6.7–100)	0.243 *	(0.238–0.255)	0.004
Diploma in Nursing	54.6 ± 15.9	53.3 (0–96.7)			
Master’s degree	66.1 ± 15.7	71.7 (43.3–80)			
Years of nursing experience					
1–5 years	54.9 ± 14.3	56.7 (30–100)	0.240 *	(0.231–0.247)	0.004
6–10 years	58.1 ± 17	56.7 (23.3–100)			
>10 years	53.6 ± 16.3	53.3 (0–96.7)			
Nationality					
Non-Saudi	54.2 ± 16.1	53.3 (0–100)	0.043 **	(0.039–0.047)	0.13
Saudi	60.2 ± 15.6	60 (23.3–100)			
Marital status					
Married	54.4 ± 15.6	53.3 (0–96.7)	0.584 *	(0.582–0.601)	<0.001
Single	57.4 ± 18.7	56.7 (20–100)			
Divorced/Widow	51.1 ± 8.9	51.7 (36.7–60)			
Children					
No	57 ± 18.1	56.7 (6.7–100)	0.148 **	(0.141–0.155)	0.09
Yes	54 ± 15.2	53.3 (0–96.7)			
Number of children					
One child	52 ± 13.5	53.3 (23.3–83.3)	0.180 *	(0.173–0.188)	0.008
Two children	54.4 ± 15.4	51.7 (0–93.3)			
Three children	61.2 ± 18.7	63.3 (26.7–96.7)			
More than three children	52.2 ± 16.5	50 (23.3–73.3)			
Department work					
Emergency room	58.9 ± 18.3	56.7 (23.3–100)	0.359 *	(0.350–0.369)	0.003
Intensive care unit Includes “MICU, SICU, PICU and NICU”	53.4 ± 14.9	53.3 (6.7–93.3)			
Labor and delivery	58.0 ± 22.8	60 (0–86.7)			
Medical	54.9 ± 13.9	56.7 (26.7–83.3)			
Obstetrics and gynecology	59.1 ± 17.6	53.3 (36.7–83.3)			
Pediatric	51.6 ± 15.1	50 (23.3–86.7)			
Surgical	54.5 ± 16.1	50 (20.0–96.7)			
Do You Prefer Night Shifts?					
No	58.1 ± 14.4	56.7 (30–100)		(0.003–0.005)	0.038
Sometimes	55.8 ± 16.2	55.0 (6.7–100)	0.005 *		
Yes	48.5 ± 16.9	43.3 (0–83.3)			
Night shifts during last month					
≤8	52.4 ± 15.4	50 (0–100)	0.003 *	(0.001–0.003)	0.19
>8	59.3 ± 16.5	56.7 (23.3–100)			

* Kruskal–Wallis test, ** Mann–Whitney test.

**Table 13:** Relationship between Intershift Recovery State Scale and sociodemographic and work-related characteristics.

Characteristics	Intershift Recovery State Scale			95% Confidence Interval	Effect Size
	Mean ± SD	Median (Range)	*p*-Value		
Age group (years)					
21–30	47.2 ± 11.6	46.7 (16.7–76.7)	0.604 *	(0.288–0.306	<0.001
31–40	49.6 ± 11	50 (20–93.3)			
41–50	50.5 ± 15.7	50 (0–100)			
>50	48.5 ± 13	50 (26.7–73.3)			
Gender					
Female	48.5 ± 12.4	50 (0–100)	0.008 **	(0.005–0.008)	0.17
Male	53.3 ± 10.7	53.3 (26.7–73.3)			
Education level					
Bachelor’s degree	48.5 ± 12.7	50 (16.7–93.3)	0.425 *	(0.423–0.442)	<0.001
Diploma in Nursing	49.9 ± 11.8	50 (0–100)			
Master’s degree	48.9 ± 15.2	53.3 (20–60)			
Years of nursing experience					
1–5 years	48.9 ± 12.8	50 (16.7–80)	0.142 *	(0.140–0.154)	0.008
6–10 years	46.6 ± 10.5	46.7 (20–70)			
>10 years	50.3 ± 12.7	50 (0–100)			
Nationality					
Non-Saudi	49.9 ± 12	50 (0–100)	0.014 **	(0.009–0.013)	0.16
Saudi	44 ± 12.7	45 (16.7–76.7)			
Marital status					
Married	49.3 ± 12.4	50 (0–100)	0.985 *	(0.951–0.960)	< 0.001
Single	48.5 ± 11.4	50 (16.7–76.7)			
Divorced/Widow	50 ± 16.3	48.3 (26.7–70)			
Children					
No	48.9 ± 13.2	50 (16.7–93.3)	0.866 **	(0.866–0.879)	0.01
Yes	49.3 ± 11.9	50 (0–100)			
Number of children					
One child	48.8 ± 10.5	48.3 (23.3–80)	0.135 *	(0.129–0.142)	0.011
Two children	50.6 ± 11.5	50 (16.7–100)			
Three children	44.7 ± 11.6	46.7 (20–63.3)			
More than three children	51.9 ± 21.2	56.7 (0–73.3)			
Department work					
Emergency room	46.8 ± 13.2	50 (16.7–70)	0.285 *	(0.275–0.293)	987,542
Intensive care unit “MICU, SICU, PICU and NICU”	50.8 ± 12.5	50 (0–93.3)			
Labor and delivery	48.4 ± 17.9	46.7 (20–100)			
Medical	46.1 ± 12.3	43.3 (23.3–80)			
Obstetrics and gynecology	47.6 ± 7.2	50 (30–56.7)			
Pediatric	50 ± 10.3	50 (36.7–73.3)			
Surgical	51.1 ± 10	50 (26.7–73.3)			
Do You Prefer Night Shifts?					
No	46.8 ± 12.0	46.7 (0–76.7)	0.106 *	(0.215–0.231)	0.002
Sometimes	49.4 ± 11.8	50 (16.7–93.3)			
Yes	52 ± 13.4	53.3 (16.7–100)			
Night shifts during last month					
≤8	50.8 ± 12.2	50.0 (16.7–100)	0.022 **	(0.020–0.026)	0.15
>8	46.4 ± 11.9	46.7 (0–70)			

* Kruskal–Wallis test, ** Mann–Whitney test.

**Table 14:** Correlation between Occupational Fatigue Exhaustion Recovery Scale and night-shift impact.

	Chronic Fatigue State Scale		Acute Fatigue State Scale		Intershift Recovery Scale	
	Correlation Coefficient	*p*-Value *	Correlation Coefficient	*p*-Value *	Correlation Coefficient	*p*-Value *
Physiological night-shift impact score	0.454	<0.001	0.412	<0.001	−0.355	<0.001
Psychological night-shift impact score	0.495	<0.001	0.375	<0.001	−0.319	<0.001
Social night-shift impact score	0.577	<0.001	0.433	<0.001	−0.355	<0.001

* Spearman correlation.

**Table 15:** Multivariate analysis of the physiological, psychological, and social impact of night-shift score.

Chronic Fatigue State Scale											
	Beta Coefficient	Unstandardized Coefficients	Standard Error	95% Confidence Interval for the Beta Coefficient	*p*-Value	VIF	Tolerance	F	R^2^	R	Regression df
Social impact of night shifts	0.438	3.9	0.7	2.5–5.2	<0.001	0.455	2.196	62	0.35	0.59	2
Psychological impact of night shifts	0.189	1.7	0.7	0.3–3.1	0.017						
**Acute Fatigue State Scale**											
	**Beta Coefficient**	**Unstandardized Coefficients**	**Standard Error**	**95% Confidence Interval for the Beta Coefficient**	***p*** **-Value**	**VIF**	**Tolerance**	**F**	**R^2^**	**R**	**Regression df**
Social impact of night shifts	0.408	2.1	0.3	1.5–2.7	<0.001	1	0.9	29.3	0.2	0.45	2
Number of night shifts during last month	0.128	4.3	2	0.4–8.2	0.034						
**Intershift Recovery Scale**											
	**Beta Coefficient**	**Unstandardized Coefficients**	**Standard Error**	**95% Confidence Interval for the Beta Coefficient**	***p*** **-Value**	**VIF**	**Tolerance**	**F**	**R^2^**	**R**	**Regression df**
Social impact of night shifts	−0.240	−0.9	0.3	−1.305	0.004	1	0.9	25.6	0.2	0.43	2
Psychological impact of night shifts	−0.223	−0.9	0.3	−1.358	0.008	1	0.9				

## Data Availability

The data presented in this study are available on request from the corresponding author. The data are not publicly available due to privacy and ethical restrictions.

## Abbreviations

The following abbreviations are used in this manuscript:

KAUH	King Abdulaziz University Hospital
OFER	Occupational Fatigue Exhaustion/Recovery
OFER-CF	Chronic Fatigue Subscale
OFER-AF	Acute Fatigue Subscale
OFER-I	Intershift Recovery Subscale
SPSS	Statistical Package for the Social Sciences
